# Antiphospholipid antibodies and atherosclerotic vascular disease: recent advances

**DOI:** 10.1007/s00296-025-06050-8

**Published:** 2025-11-29

**Authors:** Anetta Undas, Jacek Musiał, Michał Ząbczyk

**Affiliations:** 1 Department of Thromboembolic Diseases, Institute of Cardiology, Jagiellonian University Medical College, Pradnicka 80, 31-202 Krakow, Poland; 2 Krakow Centre for Medical Research and Technologies, St. John Paul II Hospital, Pradnicka 80, 31-202 Krakow, Poland; 3 2nd Department of Internal Medicine, Faculty of Medicine, Jagiellonian University Medical College, Macieja Jakubowskiego 2, 30-688 Cracow, Poland; 4 St. John Paul II Hospital, Pradnicka 80, 31-202 Krakow, Poland

**Keywords:** Antiphospholipid antibodies, Atherosclerosis, Cardiovascular risk factors, Thrombosis

## Abstract

Growing evidence indicates that antiphospholipid antibodies (aPL) and antiphospholipid syndrome (APS) are not only associated with arterial thrombosis, but also enhanced premature atherosclerosis and stenotic lesions in various vascular beds. Atherosclerotic vascular disease involving coronary, carotid, and peripheral arteries is accelerated in APS to a larger extent when systemic lupus erythematosus (SLE) or other autoimmune disorders co-exist. However, the presence of aPL by itself may enhance atherosclerosis and increase the risk of arterial thromboembolic events also in older patients who did not meet the APS classification criteria. Traditional cardiovascular risk factors in particular hypertension and hypercholesterolemia largely contribute to the development and progression of cardiovascular disease and the occurrence of its thrombotic manifestations also in patients with aPL, therefore they should be vigorously treated like in patients free of autoimmune disorders. Nevertheless, antiplatelet agents alone and in combination with vitamin K antagonists (VKAs) remain a mainstay in prevention of arterial thrombosis in APS, despite controversy around the impact of typical atherosclerotic vascular disease and its risk factors on therapeutic strategies in the presence of IgG and/or IgM aPL at significant titers. The present overview summarizes clinical evidence for the role of aPL in subclinical and clinically overt atherosclerotic vascular disease and its management.

## Introduction

Antiphospholipid syndrome (APS) is a systemic autoimmune disease characterized by the presence of autoantibodies directed against complexes of plasma proteins and negatively charged phospholipids (antiphospholipid antibodies—aPL) in particular lupus anticoagulant (LA), anticardiolipin (aCL), and anti-ß2-glycoprotein I (aß2GPI) antibodies, and clinical manifestations such as venous, arterial or microvascular thrombosis, and/or obstetric complications [1–5]. Venous thromboembolism (VTE) is observed in about 2/3 of patients with thrombotic manifestations, while arterial thromboembolism, mainly ischemic stroke or transient ischemic attacks (TIA), occurs in about 1/3 of subjects with thrombotic APS [1]. APS often coexists with other systemic autoimmune diseases, predominantly systemic lupus erythematosus (SLE) [1, 3].

In the white population, the prevalence of APS is estimated at 40–50 per 100,000 adult and the incidence at 1–2 per 100,000 per year [6]. APS occurs 5 times more frequently in women, mainly due to the APS secondary to SLE [1, 7]. A mean age at APS diagnosis has risen over the last decade up to 50 years [6, 8] and APS is increasingly diagnosed in older subjects > 50 years of age, mainly men aged above 60 [8]. aPL can be detected in 1–5% of healthy individuals and may also transiently occur during infections, mostly in low titers [9].

**Fig. 1:**
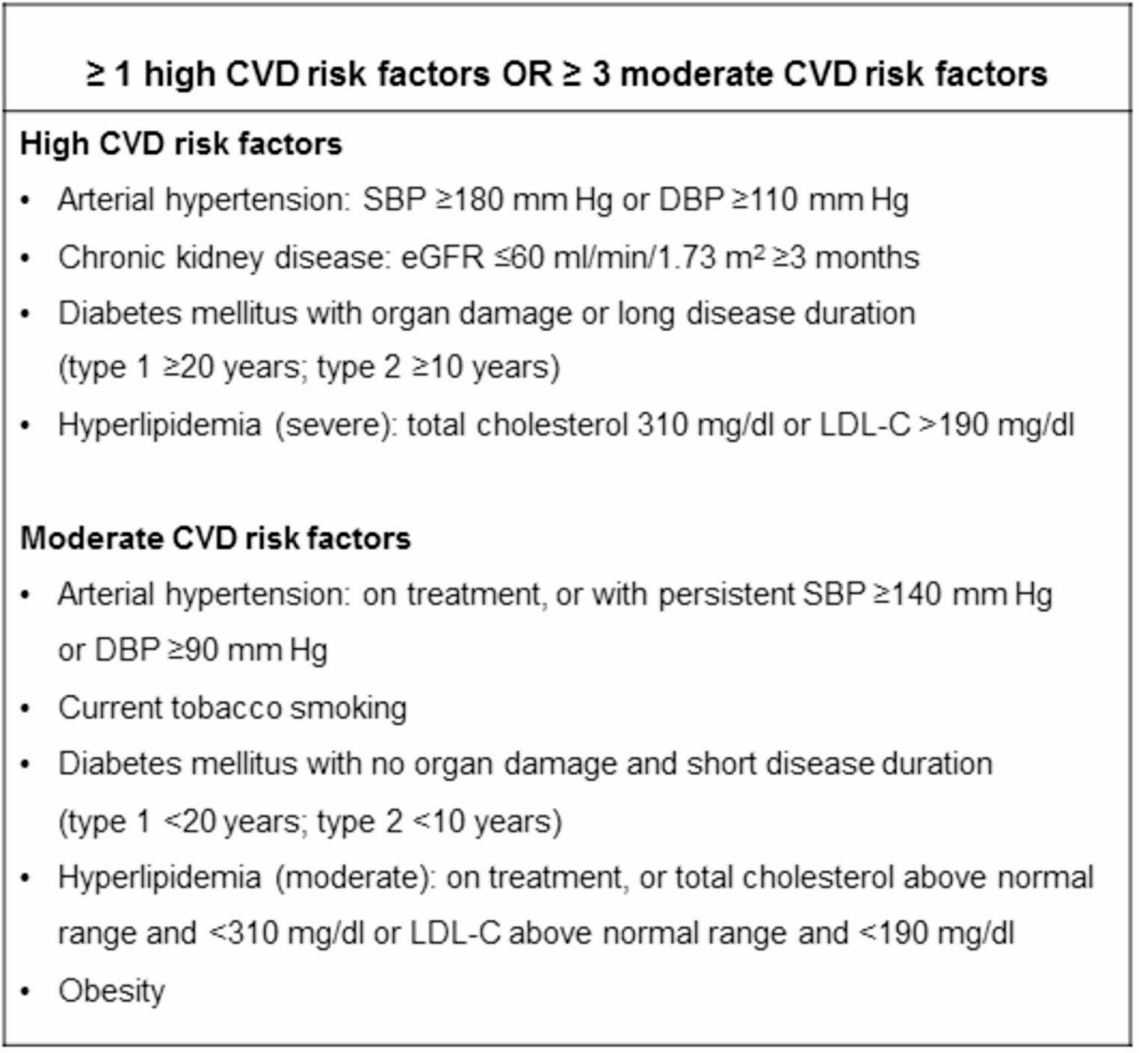
Cardiovascular risk as a criterion for antiphospholipid syndrome classification according to the 2023 ACR/EULAR guidelines [3]. *CVD* cardiovascular disease, *DBP* diastolic blood pressure, *eGFR* estimated glomerular filtration rate, *LDL-C* low-density lipoprotein cholesterol, *SBP* systolic blood pressure

The 2006 Sapporo laboratory APS criteria encompass the presence of LA and/or medium-to-high titers of IgM and/or IgG aCL and aß2GPI antibodies detected at two or more occasions at least 12 weeks apart [2]. The recent 2023 ACR/EULAR classification criteria [3] (not recommended now to establish the diagnosis of APS in everyday practice) proposed to use a different strict approach leading to a 25% reduction of subjects with arterial thrombosis classified as APS patients due to high cardiovascular disease (CVD) risk, isolated presence of IgM antibodies or both [10]. Importantly, the 2023 ACR/EULAR classification criteria [3] specified a high and moderate CVD risk (Fig. 1) based on the assumption that elevated classic CVD risk is potent enough to explain arterial thrombosis and a potential role of aPL is minor in this clinical setting. However, evidence to support such a concept is inconsistent. Generally, the risk of thrombosis is higher in the IgG than for the IgM aPL, and increases with rising titers, but the highest risk is observed in individuals with LA, aCL, and aß2GPI, i.e. the so-called “triple positivity” [11, 12]. It is important to emphasize that the diagnosis of APS in a given patient remains an individual decision of the experienced clinician due to a large clinical and laboratory heterogeneity, but aPL (without other features required to classify a subject as having APS) are largely considered clinically relevant, not only as a modulator of thrombotic events, but also a contributor to chronic vascular lesions.

It is well known that autoimmune diseases, including SLE [13] and rheumatoid arthritis (RA) [14], via the impact of inflammation and oxidative stress predispose to enhanced atherosclerosis progression with elevated risk of thromboembolic manifestations, but this link is in part related to traditional cardiovascular risk factors (age, hypertension, hypercholesterolemia, smoking, diabetes, positive family history). In this context coexisting APS appears to substantially contribute to faster development and progression of atherosclerotic vascular diseases (ASVDs) via multiple proatherogenic effects, with mounting evidence linking aPL with CVD also in individuals without any concomitant autoimmune disorders [15]. It is unclear to what extent single-positive or low-titer aPL affect atherosclerosis and its clinical manifestations in the presence and absence of conventional cardiovascular risk factors, particularly in elderly individuals. Consequently, differentiating aPL-related arterial disease from atherosclerosis, if feasible, poses a diagnostic challenge. Last but not least, evidence guiding optimal antithrombotic management in APS with concomitant atherosclerotic manifestations is limited.

The present overview summarizes data on the role of aPL in ASVDs and its manifestations with focus on the recent clinical evidence.

## Methods

This narrative review encompasses literature published mostly between January 2015 and August 2025. Key publications outside this timeframe were also considered. A comprehensive search was performed in PubMed, Web of Science, and Scopus databases using the keywords: “antiphospholipid antibodies,” “antiphospholipid syndrome,” “atherosclerotic vascular disease,” “cardiovascular disease,” “myocardial infarction,” “stroke,” “subclinical atherosclerosis,” “peripheral arterial disease,” “coronary artery disease,” “antithrombotic therapy,” and “arterial thrombosis.”

Studies included were English-language articles comprising clinical guidelines, randomized controlled trials, systematic reviews, observational studies, and translational research. References were supplemented by forward citation tracking and manual searching of reference lists from key publications. We included studies that clearly documented cardiovascular manifestations related to aPL or their mechanisms. We excluded studies unrelated to such manifestations associated with APS, along with conference abstracts, editorials and case reports.

Data were selected for relevance to mechanisms, diagnosis, prognosis, and treatment in ASVDs coexisting with APS. We screened a total of 5,432 articles, and a final analysis included 984 papers. Potential sources of bias were: isotype definition (IgG, IgM, IgA), a use of different tests (challenging standardization), anticoagulation that can influence tests results, different titers established as positive (unclear aPL positivity), and persistence of aPL.

### Mechanisms linking APS with atherosclerosis

**Fig. 2:**
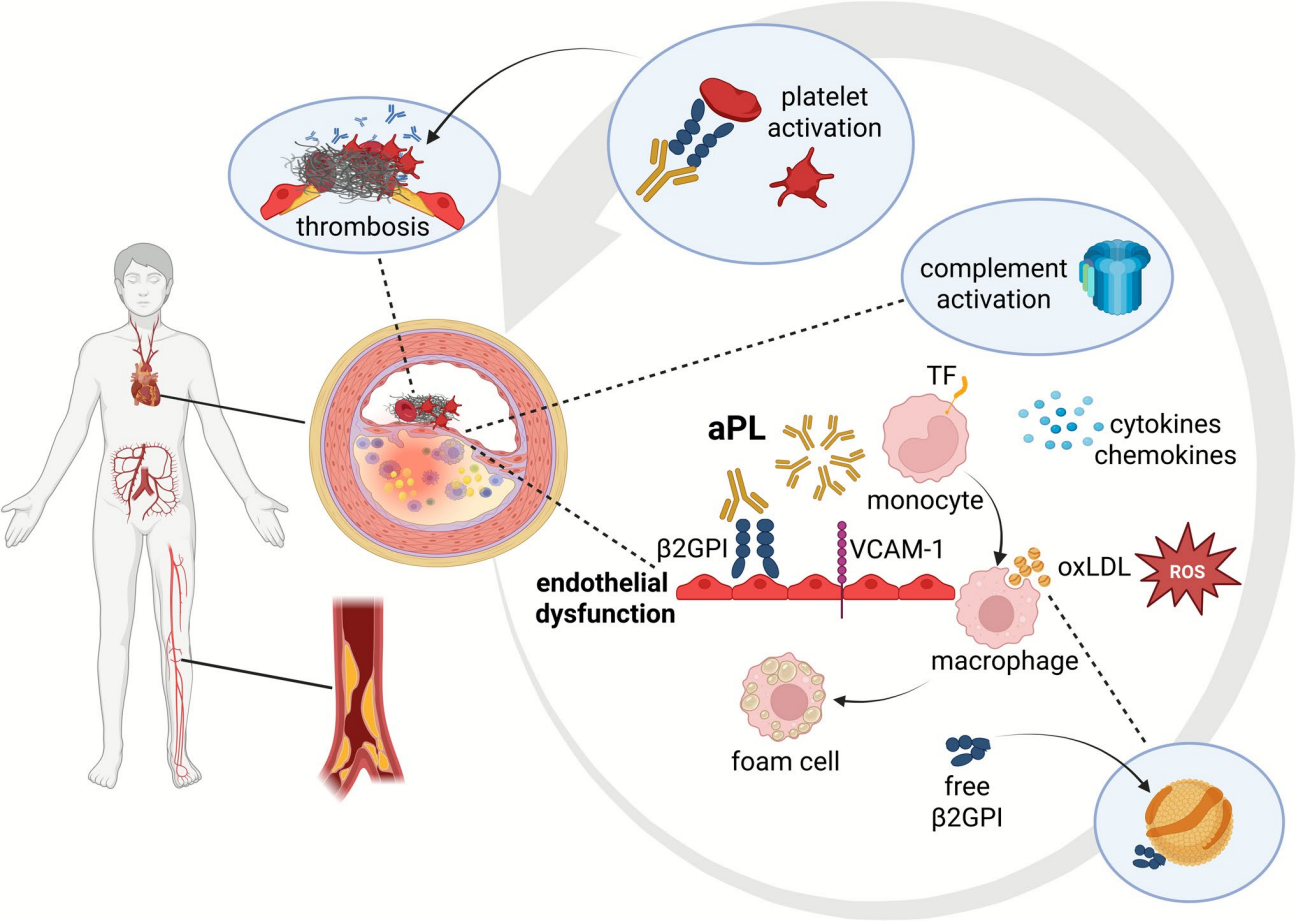
Pro-atherogenic mechanisms of antiphospholipid antibodies in vascular disease. Atherosclerotic lesions in patients with antiphospholipid syndrome (APS) are observed in coronary, carotid, and peripheral arteries. However, a strong correlation between circulating antiphospholipid antibodies (aPL) and systemic atherosclerosis, including ischemic stroke, coronary artery disease, acute myocardial infarction, and peripheral arterial disease is also reported. aPL can stimulate atherosclerosis leading to endothelial dysfunction and upregulation of β2-glycoprotein I (β2GPI) receptors on endothelial cells triggered by inflammation. aPL can also increase the expression of vascular cell adhesion molecule-1 (VCAM-1) on endothelial cells, promoting leukocytes adhesion. Enhanced oxidative stress reflected by i.a. enhanced generation of reactive oxygen species (ROS) and release of cytokines/chemokines amplify low-density-lipoprotein (LDL) oxidation and inflammation. Circulating anticardiolipin (aCL) and anti-β2GPI antibodies promote the uptake of LDL-β2GPI complexes by macrophages, leading to foam cell formation and contributing to the development of atherosclerotic plaques. Monocytes/macrophages and endothelial cells express tissue factor (TF) upon inflammation, promoting coagulation. Binding of circulating aPL to domain I of β2GPI receptors on platelets triggers platelet activation, promoting aggregation and contributing to a prothrombotic state commonly observed in APS. The thrombogenic effects of aPL also involve activation of the complement system. The membrane attack complex composed of complement components (C) 5b, C6, C7, C8, and C9, forms pores in the membranes of damaged cells, leading to their lysis, weakening the fibrous cap, and promoting rupture. Complement activation promotes inflammation and infiltration of immune cells to the vessel wall. Complement components can also bind oxidized LDL (oxLDL), enhancing its uptake. Created in BioRender. Hojda, A. (2025) https://BioRender.com/qk8kta4

Robust evidence supports the view that aPL exert several pro-atherogenic effects (Fig. 2). Circulating aPL induce an inflammatory milieu contributing to endothelial dysfunction and vascular remodeling promoting both thrombotic and atherosclerotic mechanisms [15, 16].

β2GPI can bind lipoproteins, in particular oxidatively modified low-density lipoprotein (oxLDL) and lipoprotein(a) (Lp[a]) [17]. The pro-atherogenic properties of aPL, involve formation of oxLDL-β2GPI-anti-oxLDL/β2GPI complexes [18], enhanced oxidative stress [19], release of cytokines and chemokines leading to endothelial dysfunction [16, 20], and activation of T-regulatory cells, monocytes and dendritic cells [18]. β2GPI has been reported to drive a local Th1 inflammatory response, with subsequent plaque instability favoring thrombosis [21]. Treatment of monocytes with IgG aCL antibodies altered gene expression which enhances atherosclerosis mainly when SLE coexists [9]. β2GPI drives the inflammatory response of the T helper 1 cell and T helper 17 cell in atherosclerotic plaques of patients with primary APS and SLE-related APS [21, 22]. aPL can also stimulate formation of neutrophil extracellular traps (NETs) which facilitates complement activation, inducing the release of pro-inflammatory cytokines from the endothelium and monocytes [23]. Brandt et al. [24] demonstrated that aPL colocalize with Toll-like receptors on human monocytes, activate cells in part via the clathrin/dynamin-dependent endocytic pathway and induce activation of nuclear factor kappa-light-chain-enhancer of activated B cells (NF-κB).

**Fig. 3:**
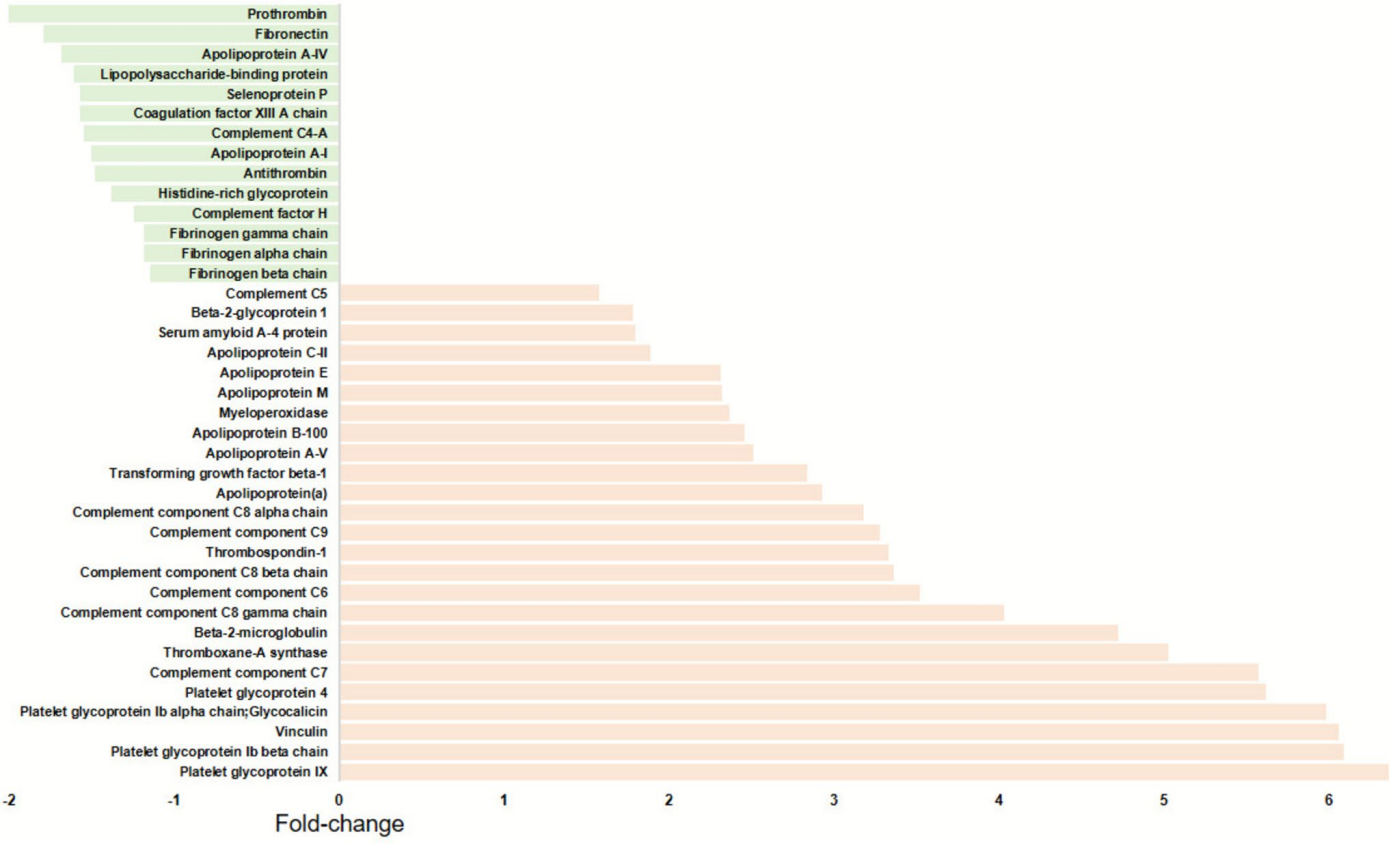
The most abundant differentially expressed proteins within plasma fibrin clots of patients with thrombotic antiphospholipid syndrome (n = 23) compared to healthy controls (n = 20) [27]. Fold change is based on differences in signal intensity data between samples. Immunoglobulin chains are not shown for clarity

Formation of more compact fibrin networks of impaired lysability has been reported to increase the risk of ASVDs [25]. Such unfavorably altered plasma fibrin clot properties can be observed in thrombotic APS with more prothrombotic phenotype following arterial thromboembolism [26]. Proteomic analysis of plasma clots from APS patients demonstrated high amounts of complement C5-C9, immunoglobulins, apolipoprotein B-100, platelet-derived proteins, thrombospondin-1, and β2GPI, together with decreased amounts of prothrombin, antithrombin, apolipoprotein A-I, and histidine-rich glycoprotein as compared to healthy subjects or patients free of APS (Fig. 3) [27]. Vikerfors et al. [28] have shown that only APS patients with previous arterial thrombosis had impaired fibrinolysis mediated by increased circulating plasminogen activator inhibitor type 1 (PAI-1) levels. Impaired fibrinolysis along with denser clot formation have also been demonstrated in APS patients with early carotid atherosclerotic lesions with a predictive value of elevated PAI-1 [29]. Taken together, increased formation of poorly lysable fibrin as a component of atherosclerotic lesions appears an important player in aPL-associated ASVDs.

### Key findings

-aPL lead to endothelial dysfunction, promote LDL and oxLDL uptake and foam cell formation.

-aPL contribute to inflammation, complement system activation, and thrombosis.

### Clinical evidence for enhanced atherosclerosis in APS

#### Subclinical atherosclerosis

Carotid intima–media thickness (IMT) is a well-established marker of subclinical atherosclerosis and a strong predictor of cardiovascular (CV) events. Surrogate markers represent flow-mediated dilation (FMD), pulse-wave velocity (PWV), and ankle-brachial index (ABI).

Cross-sectional studies have shown an increased prevalence of subclinical atherosclerosis in APS, while the presence of elevated aPL, especially aβ2GPI antibodies, triple aPL positivity and high aPL titers, has been associated with increased IMT and high risk of carotid atherosclerotic plaques (Table 1) [30].

**Table 1:** Key studies on the prevalence of the cardiovascular disease and its manifestation on the antiphospholipid syndrome

Author, year	Cervera et al. (2002) [1]		Pengo et al. (2009) [11]	Shi et al. (2017) [42]		Serrano et al. (2020) [72]		Ogata et al. (2021) [57]	Sevim et al. (2022) [41]	Qi et al. (2022) [44]	Álvarez-López et al. (2023) [59]
Thrombotic manifestations, n (%)	Initial	Cumulative	Initial	Initial	Cumulative	Initial	Cumulative	Initial	Cumulative	Initial	Initial
Arterial (all)	279 (27.9)	551 (55.1)	69 (43.1)	67 (26.6)	108 (42.9)	100 (62.3)	121 (75.6)	109 (64.9)	311 (48.4)	140 (36.6)	34 (33.0)
Ischaemic stroke	131 (13.1)	198 (19.8)	27 (16.9)	42 (16.7)	60 (23.8)	36 (22.5)	47 (29.4)	92 (54.8)	165 (25.7)	70 (18.3)	25 (24.3)
Transient ischaemic attack	70 (7.0)	111 (11.1)	15 (9.4)	11 (4.4)	11 (4.4)	8 (5.0)	10 (6.3)	–	69 (10.7)	–	–
Myocardial infarction	28 (2.8)	55 (5.5)	8 (5.0)	3 (1.2)	5 (2.0)	9 (5.6)	10 (6.3)^a^	6 (3.6)^a^	31 (4.8)	18 (4.7)^a^	2 (1.9)
Coronary bypass occlusion	–	11 (1.1)	–	–	1 (0.4)	–	–	–	–	–	–
Peripheral artery thromboembolism	19 (1.9)	103 (10.3)	15 (9.4)	11 (4.4)	22 (8.7)	10 (6.3)	12 (7.5)	5 (3.0)	30 (4.7)		7 (6.8)
Lower limb	–	43 (4.3)	15 (9.4)	–	6 (2.4)	–	–	5 (3.0)	–	23 (6.0)	7(6.8)
Upper limb	–	27 (2.7)	–	–	2 (0.8)	–	–	–	–	–	–
Digital gangrene	19 (1.9)	33 (3.3)	–	11 (4.4)	14 (5.6)	–	–	–	–	–	–
Visceral arterial thrombosis	–	58 (5.8)	3 (1.9)	–	15 (6.0)	17 (10.6)	19 (11.9)	4 (2.4)	11 (1.7)	20 (5.2)	–
Mesenteric artery	–	15 (1.5)	1 (0.6)	–	2 (0.8)	3 (1.9)	3 (1.9)	3 (1.8)	–	–	–
Splenic artery	–	11 (1.1)	–	–	3 (1.2)	–	–	–	–	–	–
Renal artery	–	27 (2.7)	2 (1.3)	–	10 (4.0)^b^	14 (8.8)	16 (10.0)^b^	1 (0.6)	–	–	–

Data are presented as number (%) and mean 6 standard deviation (SD) or range when appropriate

^a^Acute coronary episodes and/or chronic coronary syndrome

^b^Includes arterial or venous events

Several investigators have however found no differences in carotid IMT between APS patients relative to controls [31, 32]. Minimal differences ranged from 0.04 to 0.18 mm [29]. In obstetric APS typically affecting young or middle-aged women, subclinical atherosclerosis has not been observed [33]. In patients with autoimmune diseases there is a high prevalence of atherosclerotic plaques even in the absence of increased carotid IMT [34]. Several studies reported greater IMT in APS patients free of other autoimmune disorders aged > 30 years, compared with non-thrombotic controls [35].

Meta-analysis of studies published before 2023 demonstrated that patients with APS had significantly larger common carotid artery IMT (mean difference [MD] = 0.07 mm), internal carotid artery IMT (MD = 0.06 mm), carotid bifurcation IMT (MD = 0.14 mm), and had almost 4 times higher odds of detecting atherosclerotic plaques compared to controls, which was accompanied by a decreased flow and nitrate-mediated dilation in APS [36].

The increased IMT among APS patients has been linked to relatively common occurrence of well-established risk factors for atherosclerosis, as compellingly shown in the APS registries where the prevalence of hypertension, hyperlipidemia, active smoking, and metabolic syndrome was estimated at 20–35%, 20–25%, 30–40%, and 17–38%, respectively [1, 37]. The prevailing view is that traditional CV risk factors render patients with positive aPL more prone to arterial events, along with enhanced atherosclerotic lesions, being generally more prevalent in APS compared with the general population [38].

The first prospective evidence for accelerated atherosclerosis in APS associated with prior thrombosis, but free of atherosclerotic CVD events or diabetes, was published by Evangelatos et al. in 2022 [39]. During a 3-year follow-up, APS patients exhibited a 3.3-fold higher risk of new atherosclerotic plaque formation compared with matched healthy controls, similar to that in diabetic patients. Coexistence of SLE with APS increased almost eightfold the risk of plaque development as compared to those with primary APS, independently of traditional CV risk factors [39].

Recently, Evangelatos et al. [40] have demonstrated the presence of new atherosclerotic plaques in 52% of thrombotic APS patients (women, 75%) at a mean age of 44.9 years during the 7-year follow-up. APS patients, including a substantial proportion of smokers (30%) and hypertensives (27%), exhibited a threefold increased risk for plaque progression compared with controls, while 39% and 23% of patients with APS developed new plaques in carotid and femoral arteries, respectively. There were two independent risk factors associated with new atherosclerotic plaque development during follow-up, namely APS concomitant with SLE and number of traditional CV risk factors [40]. It is unclear whether a similar phenomenon can be observed in obstetric APS.

Taken together, patients with aPL should be screened for markers of enhanced atherosclerosis in particular using IMT as a well-known indicator of early ASVDs.

#### Myocardial infarction

Stroke and myocardial infarction (MI) were the most common causes of death among APS patients reported in 22.5% of cases (Table 1) [37]. MI is observed in ~ 2–5.5% of patients with APS [1, 11, 41–44], and often presents as the first manifestation of the disease (73%) [45]. Acute coronary events can result from coronary artery thrombosis with or without underlying atherosclerosis, or a microvascular injury detected by cardiac magnetic resonance imaging [46], which is also often associated with myocardial dysfunction in APS [47]. Importantly, the presence of aPL in a patient with MI does not exclude other common traditional CV risk factors, along with a history of prior ischemic stroke, rendering such individuals good candidates for APS screening [48]. Patients with primary APS who experienced acute MI or angina (women, 85%) have been reported to more frequently exhibit dyslipidemia, hypertension, and markedly increased Lp(a), along with a higher prevalence of IgM aPL [49]. Moreover, acute MI in APS is often accompanied by a high percentage of stent thrombosis and also thrombotic occlusion of coronary bypass can be observed but quite infrequently (< 1%) [1, 42]. This suggests that the follow-up of APS patients with AMI should be tight due to a considerable residual risk [50].

Of note, MI with nonobstructive coronary arteries (MINOCA) is a relatively common manifestation in patients with APS [45, 51], particularly among younger individuals [52–54], which is largely interpreted as the result of acute thromboembolic event [52]. In our experience, APS, largely single-positive, was detected in 15.5% of MINOCA patients, mostly in non-ST-segment elevation MI (27.3%) and individuals aged > 50 years (32.3%) [54].

Regarding isotypes, it is worth highlighting that evidence in favor of IgM aPL is inconsistent. It has been reported that IgM aPL are primarily found among patients with arterial events [55], but a recent study has shown that 83% of patients with arterial events were positive for IgG aCL and 63% were positive for IgG aβ2GPI antibodies [56]. Generally, the presence of aPL following acute MI, especially in younger patients, should be cautiously interpreted and managed.

#### Peripheral arterial disease

Peripheral artery disease (PAD) is far less common than peripheral venous thrombosis in APS, affecting 5–12% of patients (Table 1). Most studies on APS reported PAD affecting mainly lower limbs [11, 57–59]. Lower limb ischemia is more prevalent (~ 27–42% of cases) as compared to upper limb ischemia [1, 42].

Studies examining both the carotid and femoral sites [60] have found similar prevalence between patients with primary and secondary APS, in accordance to results by Kravvariti et al. [30] (21% versus 23%, 14% versus 18%). Using computed tomography angiography a higher prevalence of lower limb artery stenosis was observed in primary and secondary APS compared with control subjects (36% and 41% versus 23%, respectively) [61]. Symptomatic PAD confirmed by Doppler ultrasound or angiography has been associated with higher odds of aPL positivity compared with asymptomatic controls [62], which indicates that primary APS is linked to accelerated atherosclerosis in the lower limbs. Individuals with this type of APS and limb ischemia have been found to have a longer APS duration with a higher risk of arterial thrombosis, in contrast to a lower VTE risk [63]. Ankle-brachial index (ABI) below 1 can be observed in 20.5% of APS patients from 34 studies, while as few as 2.5% of controls presented such a result [36].

In a recent systematic review and meta-analysis, among patients with lower extremity artery disease, the prevalence of IgG aCL and LA was 12% and 13.3%, respectively [64], while LA was more frequent in patients with failed vs those with successful revascularization (35.8% vs 15.8%) [64].

Digital gangrene, often bilateral, can affect the upper and lower extremities [65, 66]. In most registries, it occurs in ~ 3–6% of APS patients [1, 42] and often in the context of catastrophic APS [67, 68]. Symptomatic PAD was reported in 20.5% of subjects with APL in contrast to 4.4% of those without such antibodies [69]. Occlusion of lower limb arteries can also represent a manifestation of catastrophic APS [70].

#### Other locations

Thrombosis in visceral arteries can be found in up to 6% of patients with APS [1, 44]. Mesenteric artery thrombosis has been reported in up to 2% of APS patients [1]. Chronic abdominal angina has also been described in APS. Splenic infarcts may occur in ~ 1% of APS patients [1] and this disease represents a relatively frequent cause of such infarctions in the general population [71]. Liver and pancreatic infarctions are uncommon (< 1%) [7].

#### Cerebrovascular ischemic events

In the Euro-Phospholipid cohort of 1,000 patients with APS, ischemic stroke and TIA were identified in 13% and 7%, respectively, mainly in young or middle-aged patients [1], but other studies reported even higher prevalence of positive aPL following such manifestations (Table 1). APS screening is recommended in patients with ischemic stroke aged 45–50 years or younger, regardless of the presence or absence of concomitant autoimmune disorders or signs/symptoms suggestive of APS.

The prevalence of stroke in large cohorts of APS patients ranges between 20 and 30% [1, 7, 41–44, 70], and the reported prevalence of TIA is around 10% [1, 11, 41, 72]. On the other hand, in the general population, APS can be associated with acute ischemic stroke in 20% of patients aged below 45 years [73]. Additionally, the estimated prevalence of aPL was 17.2% and 11.7% among patients with stroke or TIA, respectively, and has been associated with a five-fold higher risk for stroke or TIA compared with aPL-negative individuals in the general population [74]. In situ thrombosis and cardioembolic disease, either due to left-sided cardiac valve abnormalities (e.g. Libman–Sacks endocarditis) or, rarely, intracardiac thrombi, have been implicated in the pathogenesis of cerebrovascular ischemic events [73, 75]. APS characterizes with a broad spectrum of abnormalities on brain imaging, including large infarcts mainly affecting the middle cerebral artery and small cortical infarcts, or lacunar infarcts, along with multiple hyperintense white matter foci [76, 77]. Stroke has been shown as a common cause of mortality in APS (up to 18% of deaths) [37, 78], and the number one cause of permanent disability (commonly hemiplegia, hemiparesis) reported in up to 20% of cases [79].

Mechanisms underlying a predilection for stroke in APS with a relatively low incidence of MI remain obscure. Low thrombomodulin expression in cerebral vessels with the resultant impaired anticoagulant protein C activation is regarded as a specific prothrombotic mechanism of stroke. This implies the hemostatic imbalance leading to a prothrombotic state. aPL may have specific antineuronal ties, determining a further potential damage [80].

### Key findings

-An association between aPL and subclinical atherosclerosis exists.

-APS is associated with accelerated atherosclerosis progression.

-MI, PAD, and stroke are frequently observed in patients with aPL and APS.

#### Therapeutic implications

Mutual links of APS with ASVDs have two implications: 1. Therapies recommended in APS could affect cardiovascular morbidity, and 2. Well-established and emerging therapies used in patients free of APS can provide benefits for APS patients similar to those reported in aPL-negative patients at risk of ASVDs. However, evidence to support the 2 options is either inconsistent or of low quality.

#### Cardiovascular therapy in APS

##### Antiplatelet agents

The 2019 EULAR guidelines [81] recommend treatment with vitamin K antagonist (VKA) over monotherapy with aspirin, taking into account the thromboembolic and bleeding risk. In some high-risk cases, a combination treatment should be considered. In clinical practice, there are 2 possible scenarios: patients who experienced only arterial thrombosis, in whom VKA should be the first-choice treatment, and patients following thromboembolism and atherosclerosis mostly receive VKA and aspirin. Evaluation of the general atherosclerotic risk profile is mandatory, and the impact of the traditional risk factors should be minimized [82, 83].

##### Statins

Hydroxymethylglutaryl-coenzyme A (HMG-CoA) reductase inhibitors (statins), the potent cholesterol-lowering agents that display additional anti-inflammatory, immunomodulatory and antithrombotic effects, suppress endothelial adhesiveness induced by aβ2GPI antibodies and prevent aPL-induced vascular cell adhesion molecule up-regulation. In patients with APS, fluvastatin has been reported to inhibit IgG-mediated reactivation of factor Xa involved in the calcium flux in endothelial cells and related signaling pathways [84]. A significant reduction of proinflammatory and procoagulant parameters was reported after a 3-month treatment with fluvastatin in 41 aPL asymptomatic carriers [85]. However, statins are still used infrequently (10–30% of APS patients) [40]. Of note, during a 7-year follow-up, APS patients who achieved target LDL cholesterol had a lower carotid and femoral atherosclerotic lesion progression [40].

Statins should be prescribed according to total cholesterol and LDL cholesterol levels, and the target values of these parameters should be reached [3, 80]. In secondary prevention of arterial events, also in APS, LDL cholesterol should achieve 1.4 mmol/L or below [86]. Patient education is of key importance since adherence to statins is generally suboptimal [87]. APS is not a contraindication for PCSK9 inhibitors and should be used if a target LDL cholesterol was not achieved on high-intensity statins [88]. We have recently observed that thromboembolic events occurred less commonly in anticoagulated patients with APS receiving statins (8% vs. 50%) during a median follow-up of 53 months [89]. Collectively, despite the fact that evidence supporting the use of various statins at different daily doses in APS comes from pre-clinical or small-sized observational studies (as reviewed recently [90]), these agents represents the key class of drugs in ASVDs in the presence or absence of aPL.

#### Treatment for APS

##### Aspirin and other antiplatelet agents

Several studies support the use of aspirin (75–100 mg/day) for primary prevention of thrombotic events in aPL patients. Analysis of pooled data from 5 international cohort studies demonstrated benefits from aspirin use in the prevention of the first thromboembolic event (hazard ratio, 0.43; 95% CI, 0.25–0.75), but solely in the case of arterial manifestations, in patients with SLE and asymptomatic aPL carriers [91]. In the randomized ALIWAPAS trial [92], there was no difference between patients with aPL who received aspirin and those on aspirin combined with low-intensity warfarin in terms of thromboembolism, but the latter intervention was associated with higher bleeding risk. There is no evidence for aspirin-mediated effects on atherosclerosis progression during a 3-year follow-up [39].

The 2019 EULAR guidelines recommend primary thromboprophylaxis with 75–100 mg/d aspirin in asymptomatic aPL-positive patients without thrombotic manifestations who have a high-risk aPL profile, independently of the presence of other risk factors, and in individuals with SLE and a high-risk aPL profile [81]. However, in patients with SLE and a low-risk aPL profile, prophylactic aspirin may also be considered [81, 83]. In individuals with a low-risk aPL profile, with or without SLE, the risk stratification and the decision for implementing aspirin could be based on risk scores, for instance the GAPSS/aGAPSS (the adjusted GAPSS, aGAPSS excludes antiphosphatydyloserine-prothrombin antibodies) (Table 2), and on the general risk assessed according to the recommendations for the general population [80, 93]. It has been shown that the highest GAPSS values characterize APS patients following arterial thrombosis (mean GAPSS 12.2) and those with recurrent clinical manifestations (mean GAPSS 13.7) [94]. However, aGAPSS was not associated with atherosclerosis progression at a 3-year follow-up [39]. The current evidence does not confirm a predictive value of GAPSS or aGAPSS in patients with aPL.

**Table 2:** Global Antiphospholipid Syndrome Score (GAPSS) designed to predict cardiovascular disease in antiphospholipid syndrome [93]

Component	GAPSS
Hyperlipidemia	3
Arterial hypertension	1
aCL IgG/IgM	5
aβ2GPI IgG/IgM	4
aPS/PT IgG/IgM^a^	3
LA	4

*aß2GPI* anti-ß2-glycoprotein I, *aCL* anticardiolipin, *aPS/PT* anti-phosphatidylserine/prothrombin antibodies, *LA* lupus anticoagulant

^a^Removed from the adjusted GAPSS

The current evidence suggests that aspirin (325 mg/d) or dual antiplatelet therapy (aspirin + clopidogrel 75 mg/d) may have similar efficacy to VKAs in APS patients following a first episode of arterial thrombosis, but the results must be interpreted with caution [3].

##### Anticoagulants

The 2019 EULAR guidelines recommend treatment with VKA over aspirin alone [81]. A combination treatment (VKA + aspirin) should be considered especially when arterial thrombosis has been observed in clinically relevant atherosclerotic lesions [80]. Increased doses of VKA with an target INR above 3 can be considered for the management of arterial thrombosis, especially if standard treatment failed [80]. Based on 2 randomized controlled trials, high-dose and standard-dose warfarin (target INR of 2.0–3.0) show a similar efficacy in reducing thromboembolic events accompanied by a comparable risk of major bleeding [95]. In contrast to CVD, where low-dose rivaroxaban plus aspirin leads to reduced cardiovascular death and major adverse cardiac events, especially in high risk patients [96], such a strategy should not be considered in APS.

However, in APS associated with lower thromboembolic risk (without triple positivity) direct oral anticoagulants (DOACs) are used in practice especially in case of side effects or unstable INRs. The use of DOACs is contraindicated in patients with arterial events, due to a high risk of recurrence [3]. A meta-analysis of 4 RCTs conducted on 472 APS patients (largely triple-positive cases) showed almost 11-fold higher odds of developing stroke among individuals on rivaroxaban or apixaban (OR 10.74, 95%CI 2.29–50.38) [97, 98]. In our experience, a comparison of 66 patients treated with apixaban 5 mg bid and 86 with warfarin showed that during a median follow-up of 53 months the prevention of thromboembolism, including arterial events, can be similar in both treatment groups with a comparable risk of bleeding [89]. Whether indeed stroke risk on DOACs is high in patients with single- or double-positive APS on therapy targeting cardiovascular risk factors remains to be established.

#### Hydroxychloroquine

This common disease-modifying antirheumatic drug exerts anti-inflammatory and cardioprotective actions [99, 100]. Recently, hydroxychloroquine has been reported to reduce aPL levels and prevent thromboembolism [101]. In a systematic review and meta-analysis, although antimalarials use was associated with a lower prevalence of hypertension and diabetes in SLE, no association was found with carotid IMT progression, carotid plaque, and coronary artery calcification [102]. Hydroxychloroquine use has also failed to slow down atherosclerosis progression in carotid and femoral arteries [39, 40]. Patients with concomitant SLE and APS are the best candidates for hydroxychloroquine, regardless of any APS-related thrombotic events [3, 80, 81]. Several centers use hydroxychloroquine as an adjunct treatment with anticoagulation in triple-positive APS, and this drug generally well tolerated (annual monitoring for retinal toxicity required) is licensed in Europe in APS patients with recurrent or refractory thrombotic events [98].

### Future directions

APS is a highly heterogeneous incurable disease with high risk of arterial thrombosis and faster atherosclerosis. Much efforts are focused on therapies beyond antithrombotic agents, in particular strategies successfully administered in SLE or hematologic malignancies. Novel treatment concepts based largely on immunomodulation currently tested in clinical trials conducted in APS patients involve human monoclonal CD38-targeting antibodies (e.g. daratumumab), B-lymphocyte stimulator-specific inhibitors (belimumab), Bruton’s tyrosine kinase inhibitors (e.g. zanubrutinib), anti CD19 chimeric antigen receptor T cell, chimeric antigen receptor natural killer cell therapies, recombinant transmembrane activator, calcium modulator, and cyclophilin ligand interactor (TACI)-Fc fusion protein (telitacicept) [97]. Moreover, since β2GPI can interact with NETs, blockade of disruption of NETs and decreased complement signaling could be effective in thrombotic APS. From this perspective measurement of NETs-associated proteins e.g. citrullinated histone 3 might be useful though nonspecific for APS. Moreover, some micro-RNAs (miRNAs) have been reported to have reduced expression in neutrophils obtained from APS patients, especially miR-19b and miR-20a, that binds directly to tissue factor mRNA and suppresses its translation.

The personalized approach to APS therapy in individuals at risk of arterial ischemic events (e.g. the use of dual antiplatelet therapy, DOACs alone or in combination with antiplatelet drugs) should consider individual risk factors and include vigilant monitoring to reduce cardiovascular episodes and minimize bleeding complications inherent to anticoagulation. Anticoagulation in APS with atherosclerotic manifestations is particularly challenging and there are no data indicating that novel anticoagulants tested in stroke patients, e.g. factor XI inhibitors or glycoprotein VI inhibitors, could help suppress blood coagulation in this thromboinflammatory disease. Further phase 2 and 3 randomized trials should be designed and conducted to identify effective strategies decreasing harmful effects of aPL beyond VKA, antiplatelet agents, and statins [98].

## Conclusions

The current evidence supports the view that aPL promote atherosclerosis and its manifestations largely by amplifying the effects of established cardiovascular risk factors, particularly hypertension and hypercholesterolemia. Positive aPL predispose to early subclinical atherosclerosis as reflected among others by increased IMT, however to reduce the pace of its progression remains a challenge. Given the fact that aPL are detected in older subjects, commonly free of other autoimmune disorders, the optimal management linking therapy of autoimmune disorders and strict control of classic risk factors for MI or stroke is likely to improve clinical outcomes. The best strategy is now based on antiplatelet agents in primary prevention and VKA (with or without aspirin) in secondary prevention with concomitant administration of antihypertensive and cholesterol-lowering agents to reach guideline-recommended targets. However, personalized approaches to patients with aPL, regardless of age, are gaining increasing importance and major variables which could be used in decision making involve isotype and titers of aPL, comorbiditities, prior clinical manifestations, bleeding risk, and patient preferences [103]. Nevertheless, much experimental and clinical efforts are needed to develop personalized and more potent therapies to lower a significant risk of morbidity and mortality still observed in APS and aPL-positive patients.
